# Trends, risk factors, and outcomes of unplanned extubation in a neonatal intensive care unit: a seven-year retrospective study

**DOI:** 10.3389/fped.2025.1593335

**Published:** 2025-06-10

**Authors:** Kamal Ali, Mohammed Almahdi, Saleh S. Algarni, Saif Alsaif, Reem O. Alharbi, Maisa A. Alqahtani, Rashed Aldubaian, Malak Alsharif, Mark Castro, Abigail Esclanda, Manal Althubaiti, Mohanned Alrahili, Musaab Alshareef, Abdulaziz Homedi, Ibrahim Ali

**Affiliations:** ^1^ Neonatal Intensive Care Department, King Abdulaziz Medical City-Riyadh, Ministry of National Guard Health Affairs, Riyadh, Saudi Arabia; ^2^ King Saud Bin Abdulaziz University for Health Sciences, Riyadh, Saudi Arabia; ^3^ King Abdullah International Medical Research Center, Riyadh, Saudi Arabia

**Keywords:** unplanned, extubation, reintubation, trends, neonatal

## Abstract

**Background:**

Unplanned extubation (UE) is a critical adverse event in neonatal intensive care units (NICUs), contributing to increased morbidity, prolonged mechanical ventilation, and potential complications such as airway trauma, ventilator-associated pneumonia. This study aimed to evaluate the incidence and trends of UE over a seven-year period, identify associated risk factors, and assess clinical outcomes following these events.

**Methods:**

This retrospective observational study was conducted at the NICU of King Abdulaziz Medical City, Riyadh, from January 2018 to December 2024. Data were extracted from electronic medical records and included demographic details, ventilation-related parameters, and clinical outcomes of neonates experiencing UE. UE events were defined as the unintentional removal of an endotracheal tube during mechanical ventilation. The primary outcome was the incidence of UE per 100 ventilator days. Trends in UE rates over the seven-year study period were analyzed using linear regression. Logistic regression analysis was performed to identify predictors of reintubation following UE.

**Results:**

A total of 175 UE episodes were recorded over the study period. The annual UE rate ranged from 1.31 per 100 ventilator days in 2021 to the lowest recorded rate of 0.42 in 2024, demonstrating an overall decline. Notably, the lowest UE rate was observed in 2024, despite the highest number of ventilator days and an increase in unit capacity, coinciding with improved respiratory therapist (RT) staffing levels. Reintubation was required in 52% of cases, with 81% of those reintubated requiring immediate intervention. Lower gestational age (GA) was associated with increased odds of reintubation (OR = 0.79, 95% CI: 0.66–0.93, *p* = 0.006), as was lower birth weight (OR = 1.002, 95% CI: 1.001–1.003, *p* = 0.002). HFOV use at the time of UE was linked to a higher reintubation rate (*p* < 0.001). Duration of ventilation and length of hospital stay were significantly longer in infants who required reintubation after UE (*p* < 0.001, 0.004 respectively). Mortality prior to discharge was notably higher among neonates who required reintubation (23%) compared to those who did not (3%, *p* < 0.001). Linear regression analysis demonstrated no statistically significant trend in UE rates over the seven-year study period (*p* = 0.206).

**Conclusions:**

The study demonstrated an overall decline in UE rates over the seven-year period, with the lowest rate observed in 2024. This decline occurred despite the highest number of ventilator days and increased NICU capacity, suggesting that improvements in workforce staffing, particularly an increase in respiratory therapist coverage, contributed to enhanced patient safety. Reintubation following UE was influenced by gestational age, birth weight, and pre-extubation FiO₂ levels, emphasizing the need for improved preventive strategies. Efforts to minimize UE, including enhanced tube securement, optimization of sedation practices, and adherence to standardized care protocols, are essential for reducing associated risks and improving neonatal outcomes.

## Introduction

Unplanned extubation (UE), which refers to the accidental removal of an endotracheal tube (ETT) in patients receiving mechanical ventilation, is a common and serious adverse event occurring in neonatal intensive care units (NICUs) (1–5). A trigger tool-based study conducted in North American NICUs identified UE requiring reintubation as the fourth most frequent adverse event, following nosocomial infections, catheter infiltrations, and abnormal cranial imaging findings (2). UE rates in NICUs vary between 0.14 and 5.3 per 100 intubation days (1), significantly exceeding the rates observed in older children, at 0.61 per 100 intubation days (6). Two previously published reports from 2014 to 2017 have addressed acceptable UE rates in the neonatal population (7, 8). An American network set a goal of maintaining a UE rate below 2 per 100 ventilator days (7). Alternatively, Merkel et al. recommended that the rate should remain under 1 per 100 intubation days (8). Many NICUs track UE rates as an indicator of quality of care, with most studies aimed at reducing its occurrence setting a target of one UE per 100 ventilation days (8–12).

The seriousness of UE is primarily influenced by the need for reintubation, which has been reported to occur in 8%–100% of cases in NICUs (1), as well as the risk of short-term complications such as hypoxia, which may result in bradycardia or the need for cardiopulmonary resuscitation (CPR). UE often necessitates emergency intubation, which is less controlled and can increase the likelihood of adverse outcomes (13, 14). Repeated and urgent reintubations raise the risk of laryngeal or tracheal damage, scarring, ventilator-associated pneumonia, and lung injuries from excessive ventilation (13). Over time, these issues can result in longer hospital stays, extended periods of mechanical ventilation, and potential respiratory complications (15–19). In addition, repeated intubation attempts, can exacerbate laryngeal and tracheal edema, increase bleeding, and contribute to airway trauma, all of which may increase the likelihood of future difficult airway scenarios.

This study aimed to describe UE trends and explore their temporal relationship with unit-level changes. Additionally, we also evaluated clinical and demographic factors associated with the need for reintubation following UE. Furthermore, we sought to identify potentially modifiable practices to reduce UE risk in neonates, particularly in those requiring reintubation. By analyzing these factors, the findings provide insight into the clinical consequences of UE and its role in influencing ventilatory support needs, informing targeted interventions to enhance patient safety in the NICU.

## Methodology

This retrospective observational study was conducted at the NICU of King Abdulaziz Medical City (KAMC) in Riyadh, Saudi Arabia, over a seven-year period from January 2018 to December 2024. As a tertiary-level referral unit with advanced neonatal care services, the NICU at KAMC provided a suitable setting for assessing UE patterns in a high-risk neonatal population. This study was approved by the Institutional Review Board (IRB) of King Abdullah International Medical Research Centre (KAIMRC) (NRR25/034/3). As this was a retrospective analysis of de-identified data, the requirement for written parental consent was waived in accordance with institutional and ethical guidelines.

All neonates admitted to the NICU and placed on mechanical ventilation during the study period were included. The NICU population included in this study primarily comprised extremely preterm and very low birth weight infants. However, the unit also admits late preterm and term neonates requiring intensive care due to congenital anomalies, general surgical conditions, and other critical illness. Eligible infants were those who experienced at least one documented UE event, defined as the unintentional removal of an endotracheal tube (ETT) during mechanical ventilation, excluding planned or physician-directed extubations. Infants were excluded if they underwent planned extubation as part of clinical management, were extubated due to withdrawal of life-sustaining treatment, or were transferred from another facility without clear documentation of ventilation history. The UE rate was calculated as the number of UE events per 100 ventilator days using the formula: (Total UE events/Total ventilator days) × 100.

Data were retrospectively collected from the electronic medical records system at KAMC using a structured data collection sheet. Each UE event was identified through automated searches of patient records, daily ventilator logs, and nursing documentation, followed by manual verification. Collected data included demographic variables (gestational age, birth weight, and gender) and ventilation-related parameters (mode of ventilation at the time of UE, duration of mechanical ventilation prior to UE, and sedation use at the time of UE). UE-specific details recorded included the number of UE episodes per patient, and clinical events preceding the UE event, such as routine care, ETT retaping, suctioning, procedures, position changes, and transport. The study also examined key outcomes related to UE, including the need for reintubation, time to reintubation, and frequency of single vs. multiple UE events. Adverse events following reintubation, including multiple intubation attempts, new chest radiograph changes, positive tracheal aspirates, air leaks, stridor, and cardiopulmonary arrest, were assessed. In our study, multiple intubation attempts were defined as ≥2 attempts required to successfully reintubate the infant during a UE-related reintubation event. The impact of UE on NICU length of stay and the duration of mechanical ventilation also evaluated.

### Unplanned extubation prevention measures and monitoring strategies

To minimize UE events, a structured set of interventions were implemented in the NICU. Standardized ETT stabilization was enforced through uniform securing supplies, including designated taping materials. Additionally, a Two-Person Care Protocol was mandated for critical procedures such as patient repositioning, ETT retaping, and transportation, ensuring that one provider-maintained airway stability while the other performed necessary interventions. The use of bed tree handles was restricted to cases requiring Intraventricular Hemorrhage (IVH) bundle care to minimize excessive handling. A difficult airway alert system was implemented to flag high-risk neonates on bedside monitors and care plans, ensuring early recognition and preparedness during airway management. An agitation assessment protocol was established using the Withdrawal Assessment Tool-1 (WAT-1) to monitor signs of agitation and withdrawal in intubated neonates, enabling timely adjustment of sedation and analgesia.

Training initiatives reinforced best practices, requiring all new NICU staff to complete mandatory airway management competencies. Patient reassessments were incorporated into routine clinical rounds to ensure adherence to UE prevention strategies.

To facilitate event tracking and compliance monitoring, a structured documentation system was implemented. All UE events were recorded in the Electronic Patient Records (EPR) respiratory report, with additional documentation in the Safety Reporting System (SRS) to ensure standardized reporting and root cause analysis. An unplanned Extubation reporting form systematically tracked UE occurrences, enabling trend analysis and quality improvement initiatives. Monitoring metrics included UE rates, associated complications, and the relationship between ventilator days and UE events. Process measures focused on protocol compliance, completion of required competency training, and accuracy of documentation. All core practices aimed at UE prevention, were established at the start of the study period and remained in place throughout the entire 7-year duration.

### NICU expansion and staffing adjustments in 2024

During the study period, a major structural transition occurred in 2024 with the relocation to a newly built NICU, increasing ICU cot capacity from 40 to 50. This expansion was accompanied by an increase in respiratory therapist (RT) staffing, from 3 therapists managing 40 cots in the previous unit to 6 therapists covering 50 cots in the new NICU. These changes enhanced unit capacity and resource allocation, potentially influencing patient management and clinical outcomes, including UE monitoring and prevention.

### Statistical analysis

All statistical analyses were conducted using IBM SPSS Statistics (version 26, IBM Corp., Armonk, NY, USA). Descriptive statistics were used to summarize baseline characteristics, ventilation-related variables, and UE outcomes. The Shapiro–Wilk test was used to assess normality, and all continuous variables were found to be non-normally distributed. Therefore, these variables were reported as medians with interquartile ranges (IQR). Categorical variables were expressed as frequencies and percentages.

Comparisons between neonates who required reintubation following UE and those who did not were performed using the Mann–Whitney *U* test for continuous variables and chi-square or Fisher's exact tests for categorical variables, as appropriate. To identify independent risk factors for UE and the need for reintubation, a multivariate logistic regression analysis was conducted. Odds ratios with 95% confidence intervals were reported for each predictor variable. Variables included in the multivariable logistic regression were selected based on clinical relevance and significance in univariate analysis. Birthweight was retained as an indicator of underlying physiological immaturity and baseline illness severity, which may influence the course of respiratory support and the likelihood of reintubation, even at later postnatal ages.

Trends in UE rates over the seven-year study period were analyzed using linear regression, with the year of occurrence as the independent variable and UE rate per 100 ventilator days as the dependent variable. A *p*-value of less than 0.05 was considered statistically significant for all analyses.

## Results

A total of 175 episodes of UE occurred during the seven-year study period in 141 infants. Among these, 127 were single UE episode, while 48 were multiple UE events. The median gestational age (GA) of the study population was 28 weeks, IQR [25,34], with a median birth weight (BW) of 1,003 g, IQR [683, 1,977]. The median postnatal age at the time of UE was 30 days, IQR [11.5, 53], and the duration of mechanical ventilation prior to the UE event was 10 days, IQR [4, 30]. Sixty-four percent of the infants in the study were males.

**Figure 1:**
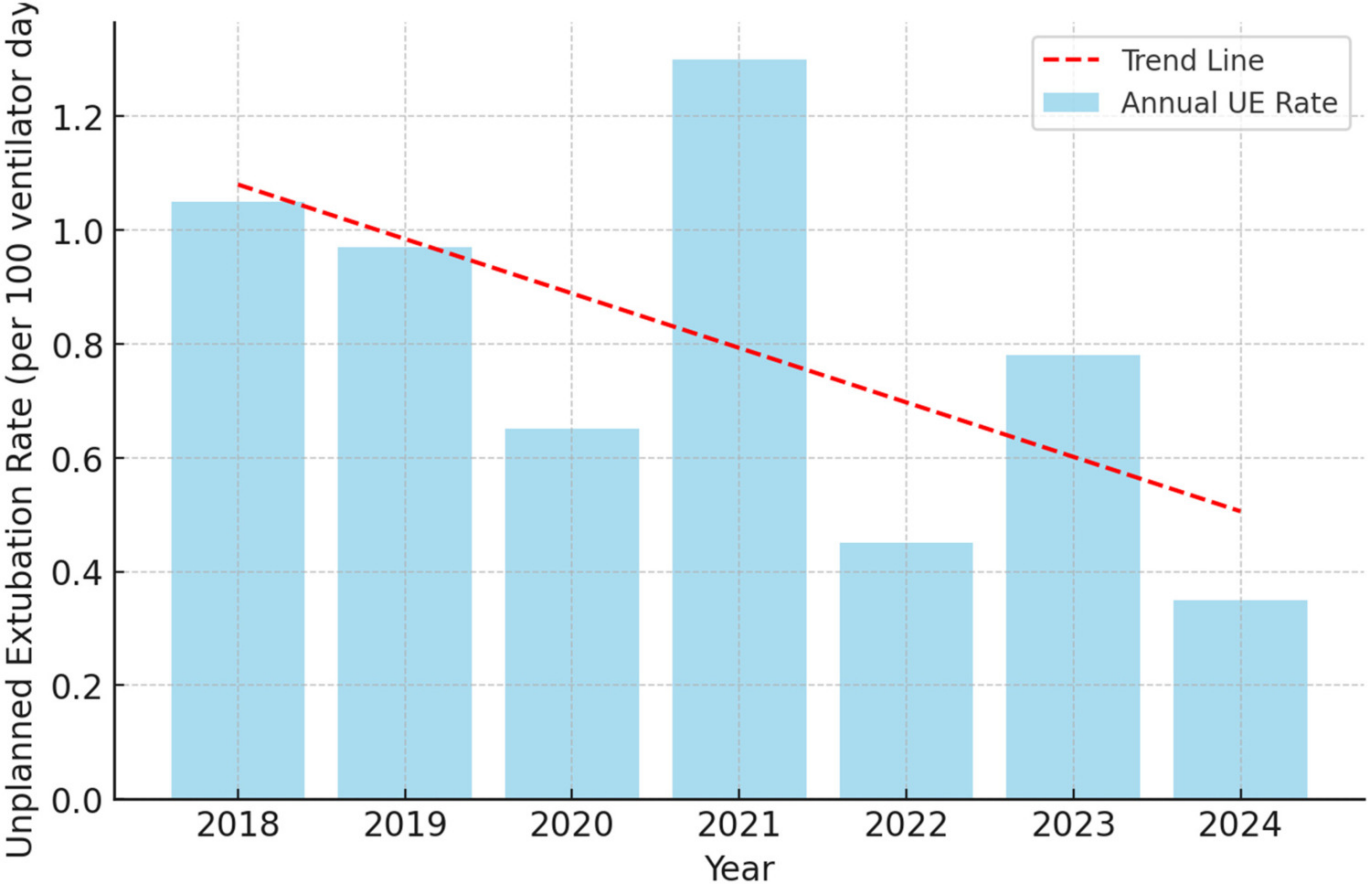
Annual unplanned extubation rate (2018–2024).

Figure 1 presents the annual UE rates per 100 ventilator days from 2018 to 2024. The blue bars represent the annual UE rate, while the red dashed line indicates the overall trend over the study period. The UE rate remained above 1.0 per 100 ventilator days in 2018 and 2021, with the highest rate recorded in 2021. A decrease was observed in 2020, followed by an increase in 2021. In 2022, the rate declined, and a slight increase occurred in 2023 before reaching the lowest recorded rate in 2024 (Figure 1).

Table 1 presents annual UE rates per 100 ventilator days from 2018 to 2024, along with the total number of ventilator days and UE events for each year. The number of ventilator days varied across the study period, with an increase in 2024 following the expansion of NICU capacity. The total number of UE events fluctuated year to year, with the highest recorded in 2021 and the lowest in 2022. The annual UE rate showed variation over time, with a notable decline in 2024, coinciding with the increase in NICU capacity and staffing (Table 1).

**Table 1:** Annual unplanned extubation rates.

Year	Ventilator days	Total unplanned extubation (UE)	Annual UE rate (per 100 ventilator days)
2018	3,029	32.0	1.06
2019	3,165	30.0	0.95
2020	2,273	16.0	0.70
2021	3,050	40.0	1.31
2022	2,887	15.0	0.52
2023	2,324	21.0	0.90
2024	4,995	21.0	0.42

Table 2 summarizes the characteristics, management, and outcomes of 175 episodes of UE. Reintubation was required in 52% of cases, while 48% of infants did not require further airway intervention. The majority of UEs (90%) occurred while the infants were on conventional mechanical ventilation, whereas 10% took place during high-frequency oscillatory ventilation (HFOV). Among the 91 cases that required reintubation, 81% were reintubated immediately, 7% within the first hour, another 7% within 12 h, 1% between 12 and 24 h, and 4% between 24 and 48 h.

**Table 2:** Characteristics, management, and outcomes of unplanned extubation events.

Variable	Outcome	Percentage
Reintubation following UE (*n* = 175) (%)	Reintubation	52
	No reintubation	48
Mode of ventilation at the time of UE (*n* = 175) (%)	Conventional ventilation	90
	HFOV	10
Time to Reintubation (*n* = 91) (%)	Immediately	81
	Within 1 h	7
	Within 12 h	7
	12–24 h	1
	24–48 h	4
Frequency of UE (*n* = 175) (%)	Single UE	73
	Multiple UE	27
Use of sedation at the time of UE (*n* = 175) (%)	Sedation	39
	None	61
Events prior to UE (*n* = 175)	Routine care	11
	Retaping of ETT	17
	Suction of ETT	14
	Procedure	11
	Position change	5
	Transport	1
	None	41
Adverse events following reintubation (*n* = 91) (%)	Multiple intubation attempts	38
	New Chest radiograph changes	9
	Positive Tracheal Aspirates	16
	Air leak	1
	Stridor	2
	Cardiopulmonary arrest	1
	None	33

UE, unplanned extubation; HFOV, high frequency oscillatory ventilation; ETT, endotracheal tube.

Regarding the frequency of UE events, 73% were single occurrences, while 27% involved multiple extubations in the same patient. Sedation was used in 39% of cases, whereas 61% occurred in the absence of sedation. Identified preceding events included routine neonatal care (11%), retaping of the endotracheal tube (17%), endotracheal suctioning (14%), a medical procedure (11%), patient repositioning (5%), and transport (1%). Notably, 41% of cases had no identifiable preceding event.

Adverse events following reintubation were recorded in 91 cases. Among these, 38% required multiple reintubation attempts, 9% exhibited new chest radiograph changes, and 16% had positive tracheal aspirates. Air leaks and post-reintubation stridor were reported in 1% and 2% of cases, respectively. One case of cardiopulmonary arrest (1%) was observed following reintubation. However, 33% of reintubated infants did not experience any immediate complications (Table 2).

**Figure 2:**
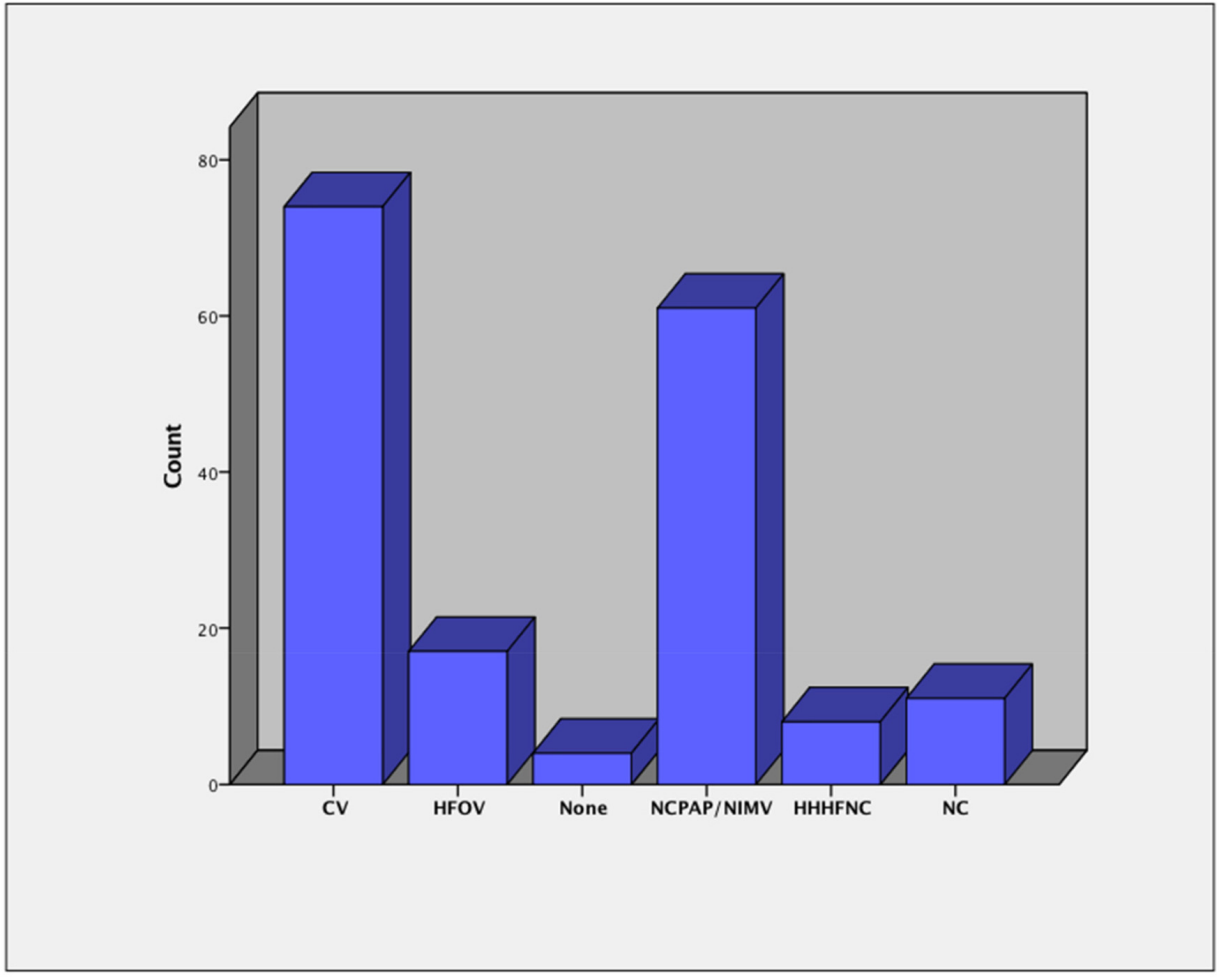
Patterns of respiratory support following 175 episodes of unplanned extubation.

Figure 2 illustrates the patterns of respiratory support following the episodes of UE. Among the 175 episodes of UE, conventional ventilation (CV) was the most frequently used mode of respiratory support post-UE, accounting for 74 cases (42%). HFOV was used in 17 cases (10%). Non-invasive respiratory support was implemented in a significant proportion of cases, with nasal continuous positive airway pressure (NCPAP) or non-invasive mechanical ventilation (NIMV) being the most common, used in 61 cases (35%). Heated humidified high-flow nasal cannula (HHHFNC) was used in 8 cases (5%), and low-flow nasal cannula (NC) was applied in 11 cases (6%). In 4 cases (2%), no respiratory support was documented following UE (Figure 2).

Table 3 compares clinical characteristics and respiratory parameters between neonates who required reintubation (*n* = 91) and those who did not (*n* = 84) following UE. There were no statistically significant differences in GA between the two groups (*p* = 0.133). Similarly, BW did not differ significantly between groups (*p* = 0.083). Postnatal age at the time of UE was significantly higher in the reintubation group (38 days, IQR [14, 58]) compared to 28 days, IQR [7.8, 46.8], in those who did not require reintubation (*p* = 0.048). Duration of ventilation prior to UE did not significantly differ between the groups (*p* = 0.411). FiO₂ requirements were significantly higher in neonates who required reintubation both before (35%, IQR [29, 45] vs. 28%, IQR [22, 30], *p* < 0.001) and after UE (35%, IQR [30, 50] vs. 30%, IQR [22, 35], *p* < 0.001). HFOV was used at the time of UE in 19% of the reintubation group compared to none in the no-reintubation group (*p* < 0.001). Among infants who experienced UE while on HFOV, no specific procedures or interventions were documented at the time of extubation. Sedation use at the time of UE was more frequent among those requiring reintubation (47% vs. 30%, *p* = 0.013). Duration of mechanical ventilation was significantly longer in the reintubation group (49, IQR [27, 74]) vs. 28, IQR [7, 44], *p* < 0.001) compared to those who did not require reintubation. Length of hospital stay was also extended in those requiring reintubation, with a median of 111 days (IQR [71, 159]) vs. 86 days (IQR [37, 118], *p* = 0.004). Mortality prior to discharge was notably higher among neonates who required reintubation (23%) compared to those who did not (3%, *p* < 0.001) (Table 3).

**Table 3:** Comparison of neonates requiring reintubation vs. those who did not after unplanned extubation.

Variable	Reintubation (*n* = 91)	No reintubation (*n* = 84)	*P* value	Total cohort (*n* = 175)
Gestational age (weeks)	27 [24,33]	29 [25,35.8]	0.133	28 [24,34.5]
Birthweight (grams)	920 [655,1,825]	1,027 [750,2,300]	0.083	980 [680,2,120]
Postnatal age (days)	38 [14,58]	28 [7.8,46.8]	0.048	33 [11,52]
Duration of ventilation prior to UE (days)	12 [4,30]	9.5 [3.2,29.5]	0.411	11 [3.6,30]
FiO2 prior to UE	35 [29,45]	28 [22,30]	<0.001	32 [25,40]
FiO2 post UE	35 [30,50]	30 [22,35]	<0.001	33 [26,45]
HFOV at UE (%)	19	0	<0.001	10.9
Sedation at UE (%)	47	30	0.013	44
Duration of mechanical ventilation (days)	49 [27,74]	28 [7,44]	<0.001	40 [17,65]
Length of hospital stay (days)	111 [71,59]	86 [37,118]	0.004	96 [52,140]
Mortality prior to discharge (%)	23	3	<0.001	13

UE, unplanned extubation; HFOV, high frequency oscillatory ventilation; FIO_2_, fraction of inspired oxygen.

A linear regression analysis was conducted to examine the trend in annual UE rates over time, with the year of occurrence set as the independent variable and the annual UE rate as the dependent variable. The model yielded an unstandardized coefficient (*B*) of −0.079 (95% CI: −0.218 to 0.061), indicating a negative association between time and UE rate. The constant term (*B* = 1.151, *p* = 0.005) represents the estimated UE rate at the baseline year (2018). The standard error for the time variable was 0.054, and the standardized coefficient (Beta) was −0.544. However, the association was not statistically significant (*p* = 0.206), meaning that while the trend suggests a decline in UE rates over the years, this decrease does not reach statistical significance (Table 4).

**Table 4:** Linear regression analysis of UE rates over time.

Variable	*B* (unstandardized coefficient)	Std. error	Beta (standardized coefficient)	*p*-value	95% confidence interval (lower–upper)
Constant	1.151	0.242	—	0.005	0.529–1.774
Year (2018–2024)	−0.079	0.054	−0.544	0.206	−0.218–0.061

A binary logistic regression analysis was conducted to identify factors associated with the likelihood of reintubation following UE. The model included GA, BW, postnatal age at UE, duration of ventilation prior to UE, and the use of sedation at the time of UE. The results show that GA had a significant negative association with reintubation (*B* = −0.239, *p* = 0.006), indicating that for each additional week of gestation, the odds of reintubation decreased by approximately 21.3% (OR = 0.787, 95% CI: 0.664–0.934). Similarly, BW was significantly associated with lower odds of reintubation (*B* = 0.002, *p* = 0.002), with an increase of 1 gram in BW associated with a 0.2% decrease in the odds of reintubation (OR = 1.002, 95% CI: 1.001–1.003). Postnatal age (*B* = 0.002, *p* = 0.657), duration of ventilation prior to UE (*B* = −0.001, *p* = 0.922), and sedation at the time of UE (*B* = 0.320, *p* = 0.396) were not significantly associated with reintubation (Table 5).

**Table 5:** Logistic regression analysis for reintubation following unplanned extubation.

Variable	*B*	S.E.	Wald	Sig.	OR	95% CI for OR (lower–upper)
Gestational Age (GA)	−0.239	0.087	7.552	0.006	0.787	0.664–0.934
Postnatal Age	0.002	0.005	0.198	0.657	1.002	0.992–1.013
Birth Weight (BW)	0.002	0.001	9.166	0.002	1.002	1.001–1.003
Ventilation Prior to UE	−0.001	0.011	0.010	0.922	0.999	0.978–1.020
Sedation at UE	0.320	0.377	0.720	0.396	1.377	0.657–2.886

UE, unplanned extubation; OR, odds ratio; CI, confidence interval.

## Discussion

Unplanned extubation (UE) is a recognized patient safety concern in neonatal intensive care units, with implications for respiratory stability, length of stay, and overall morbidity. Our study aimed to describe UE trends and explore their temporal relationship with unit-level changes. We also evaluated clinical and demographic factors associated with the need for reintubation following UE. Additionally, we sought to identify potentially modifiable practices to reduce UE risk in neonates, particularly in those requiring reintubation.

The analysis of UE rates at the Neonatal ICU of KAMC-Riyadh from 2018 to 2024 revealed annual rates ranging from 0.42 to 1.31 per 100 ventilator days, which aligns favorably with previously suggested acceptable thresholds. According to Mbi Ndakor et al., an American network adopted a target UE rate of less than 2 per 100 ventilator days in 2017, following the Vermont Oxford Network's Collaborative on Controversies in Respiratory Care (7). Our findings consistently fall well below this threshold across all years of observation. However, when compared to the recommendation by Merkel et al. in 2014, which proposed a stricter acceptable rate of less than 1 per 100 ventilator days (8), our UE rates exceeded this standard in three of the seven years, with 2021 showing the highest rate of 1.31. Compared to an Australian study conducted in 2018, which reported a UE rate of 4.75 per 100 ventilator days using a dedicated audit tool among 182 neonates (12), our recorded rates were significantly lower. In a recent study from the United States, the rate of UE was initially 0.92 per 100 ventilator days in an infant cardiac NICU. Following the implementation of an airway bundle, the rate dropped to 0.45 per 100 ventilator days, followed by a sustained 480-day period without any UE events (20). These comparisons highlight that, while our unit performs better than the rates reported in the Australian study, there is room for improvement to meet the stricter standard proposed by Merkel et al. and the recently reported UE rates from the United States study. These comparisons highlight the importance of continuous monitoring and targeted interventions to ensure UE rates remain within acceptable limits and trend toward further reductions over time.

During 2024, the total number of ventilator days was the highest across the seven-year study period, corresponding to the increase in ICU cot capacity following the transition to a newly built unit. Despite this rise in ventilator days, the UE rate was the lowest recorded during the study period. This coincided with the doubling of RT staffing, which increased from three therapists covering 40 cots in the old unit to six therapists for 50 cots in the new NICU. The enhanced RT coverage may have contributed to improved ventilator management, more consistent airway monitoring, and better adherence to extubation protocols, ultimately reducing the incidence of UE even in the context of higher patient acuity and mechanical ventilation demand. These findings suggest that staffing adjustments and resource allocation play a crucial role in optimizing respiratory care and preventing UE in the NICU setting. Although physician coverage and the 1:1 nursing ratio remained constant throughout the study period, the physical layout of the NICU improved significantly following the expansion. The redesigned unit included two-bed intensive care rooms and six single-bed isolation rooms, all integrated into a centralized monitoring system serving up to 25 ITU cots. These structural enhancements likely facilitated better patient visibility and earlier detection of extubation risk, further supporting the reduction in UE rates observed in 2024. However, while the decline in UE rates observed following NICU expansion and increased RT staffing is encouraging, it is important to recognize that our analysis captures only the immediate post-expansion period. The sustainability of these improvements likely depends on the ongoing availability of adequate staffing levels and resources. Future longitudinal evaluations are needed to determine whether the observed reduction in UE rates can be maintained over time with stable workforce support.

Our linear regression analysis demonstrated a general downward trend in UE rates over the study period; however, this trend did not reach statistical significance. The year-to-year variability observed in UE rates likely reflects the influence of multiple factors, including shifts in clinical practices, patient demographics, and unit-level operational changes. While monthly or quarterly run charts were not included in this analysis, UE trends were interpreted in the context of major system-level interventions implemented throughout the study period. Future research using prospective data collection and time-series analysis methods may provide a more precise understanding of the relationship between specific interventions and changes in UE incidence.

In our cohort, the most common events preceding UE were ET tube retaping and suctioning, followed by routine care and procedures such as PICC line insertion. These events often involve multiple caregivers and require coordinated actions, increasing the risk of UE. While a two-person approach for ET retaping is in place, there is a need to strengthen adherence through targeted RT training and routine audits of securement practices. These findings highlight the influence of human factors—such as communication, task coordination, and situational awareness—on UE occurrence. Structured approaches including pre-procedure briefings, airway management checklists, and simulation-based training may support safer practice. Emphasizing both technical and non-technical skills in RT and nursing education remains essential, especially during high-risk procedures.

Our study showed that UE in the NICU is associated with notable clinical consequences. Over half of the cases (52%) required reintubation, and the majority of these (81%) occurred immediately, suggesting inadequate respiratory stability following UE. Additionally, 27% of neonates experienced multiple UE episodes, indicating potential issues with securement or care processes. In 61% of cases, sedation was not used, highlighting agitation and patient movement as major contributing factors. Notably, 41% of events had no identifiable preceding trigger, reflecting the unpredictable nature of UE and the need for consistent monitoring and securement. Strengthening airway management strategies—such as adopting two-person ET tube stabilization during high-risk procedures and standardizing securement techniques—can help reduce UE risk. In our NICU, sedation practices include continuous fentanyl infusion guided by protocolized assessments using the Withdrawal Assessment Tool-1 (WAT-1), and PRN use of fentanyl or midazolam for agitated or high-risk neonates on prolonged ventilation. Adjustments are made based on individual clinical status and procedural requirements.

Adverse events following reintubation were common, with 38% of cases requiring multiple intubation attempts, increasing the risk of airway trauma and long-term complications. The risk of difficult intubation may increase with each reintubation attempt due to progressive airway trauma and mucosal edema, further complicating airway management and heightening the risk of cardiopulmonary instability. Additionally, 16% of neonates developed positive tracheal aspirates, suggesting a potential link between UE, reintubation, and an increased risk of ventilator-associated infections. In our cohort, cardiopulmonary resuscitation was required in 1% of UE events. Although this reflects our local experience, prior studies have reported higher rates ranging from 1% to 6%, highlighting the potential severity of UE and the need for prompt recognition and management (21).

Emergency intubations following UE have been associated with a significant increase in adverse events compared to elective or urgent intubations. Studies have shown that emergency reintubation can lead to multiple complications, including tracheal and pulmonary injury, as well as a higher risk of intraventricular hemorrhage (22, 23). Additionally, repeated intubation attempts have been identified as a risk factor for severe subglottic stenosis in NICU graduates (24)*.* Cardiovascular instability has also been reported in 20% of UEs among neonates and children admitted to NICU, PICU, or cardiac ICU, further highlighting the serious consequences of UE-related reintubation (25).

We have shown that the need for reintubation was more frequent among those on HFOV at the time of UE, suggesting a greater severity of respiratory compromise in this group. The increased oxygen requirement in neonates who required reintubation reflects their greater respiratory instability, making them more prone to post-extubation failure. Additionally, sedation use was more common among those who required reintubation, possibly reflecting differences in clinical management approaches or its impact on respiratory effort. These findings highlight that neonates with greater respiratory support needs, particularly those on HFOV or with elevated oxygen requirements, are at increased risk for reintubation following UE, emphasizing the need for careful monitoring and preventive strategies in this high-risk population. To further explore the clinical trajectory at the time of UE, we observed that infants on HFOV had a significantly higher reintubation rate, suggesting that these neonates were likely in a more acute phase of illness. Among those on conventional ventilation, while detailed staging of illness (acute vs. weaning phase) was not systematically recorded, the relatively lower oxygen requirements and sedation rates suggest that many were in a more stable or weaning phase at the time of UE. Furthermore, in our study, neonates requiring reintubation had prolonged durations of mechanical ventilation and hospital stay, indicating greater respiratory instability and increased healthcare resource utilization. The higher mortality rate in this group further emphasizes the serious implications of UE-related reintubation.

To further explore independent predictors of reintubation, we conducted a logistic regression analysis, which confirmed that lower gestational age and birth weight were significantly associated with an increased likelihood of reintubation following UE. Notably, postnatal age, duration of ventilation before UE, and the presence of sedation infusions were not significant predictors in the regression model. While sedation infusions were more commonly used among infants who required reintubation in univariate analysis, this did not independently impact reintubation risk when adjusting for other factors. Importantly, all intubation procedures in our unit were performed under procedural sedation using fentanyl, and the reference to sedation in our analysis pertained exclusively to ongoing sedative infusions for ventilated infants.

This study has several strengths that contribute to the existing body of knowledge on UE in neonatal intensive care. First, the study was conducted over a seven-year period in a high-volume tertiary NICU, allowing for a robust evaluation of UE incidence, trends, and risk factors across a large and diverse patient population. The extensive dataset enhances the generalizability of the findings, particularly for NICUs with similar care models and patient demographics. Second, the study employed a rigorous methodology for data collection, including automated searches, manual verification, and structured documentation in the electronic patient records system, ensuring high data accuracy. The use of multivariate logistic regression allowed for the identification of independent predictors of reintubation following UE, providing valuable insights into high-risk neonates who may require closer monitoring and proactive interventions. Additionally, the study analyzed trends over time, offering important information on the impact of evolving clinical practices, staffing adjustments, and unit expansions on UE rates and patient outcomes. The findings highlight the importance of resource allocation, particularly the increased RT staffing, in reducing UE rates despite a rise in ventilator days following the NICU expansion. Despite these strengths, certain limitations should be acknowledged. As a retrospective observational study, the findings are subject to inherent limitations such as potential documentation inconsistencies and missing data, which could impact the accuracy of some variables. Additionally, while efforts were made to control for confounding factors, residual confounding may still exist, particularly in assessing the precise clinical conditions leading to UE. The study was conducted at a single center, which may limit the generalizability of findings to NICUs with different care protocols and patient populations. Another limitation is the lack of long-term neurodevelopmental follow-up data, which could provide additional insights into the broader impact of UE on neonatal outcomes. Finally, an important limitation is the lack of systematically recorded data on extubation failure over the 7-year period. As a result, we were unable to assess temporal trends or determine whether evolving clinical practices may have influenced extubation readiness and outcomes. Inclusion of this metric could have provided valuable insights into the interplay between UE prevention efforts and decisions surrounding the timing of extubation. Future prospective, multicenter studies with standardized reporting, long-term outcome tracking, and inclusion of extubation failure metrics are needed to further evaluate the effectiveness of UE prevention strategies and optimize neonatal respiratory care.

## Conclusion

This study provides a comprehensive assessment of unplanned extubation (UE) rates, associated risk factors, and clinical outcomes over a seven-year period in a tertiary-level NICU. Although a general decline in UE rates was observed, year-to-year variability indicates the continued need for targeted quality improvement initiatives. Our findings highlight that neonates with lower gestational age, lower birth weight, and higher pre-extubation FiO₂ levels are at greater risk of requiring reintubation following UE, reflecting higher respiratory instability. To minimize UE and its complications, preventive strategies remain essential. These include the implementation of an airway management bundle, standardized tube securement protocols, focused staff education, and structured sedation practices. Continued surveillance and targeted interventions such as these are vital to sustaining improvements and optimizing respiratory care in ventilated infants.

## Data Availability

The data analyzed in this study is subject to the following licenses/restrictions: The data used in the study is available from the corresponding author on a reasonable request. Requests to access these datasets should be directed to Dr Kamal Ali alika@ngha.med.sa.
